# An E2E Network Slicing Framework for Slice Creation and Deployment Using Machine Learning

**DOI:** 10.3390/s23239608

**Published:** 2023-12-04

**Authors:** Sujitha Venkatapathy, Thiruvenkadam Srinivasan, Han-Gue Jo, In-Ho Ra

**Affiliations:** 1 TIFAC-CORE in Cyber Security, Amrita School of Engineering, Amrita Vishwa Vidyapeetham, Coimbatore 641112, Tamil Nadu, India; v_sujitha@cb.amrita.edu; 2 School of Electrical Engineering, Vellore Institute of Technology, Vellore 632014, Tamil Nadu, India; thiruvenkadam.s@vit.ac.in; 3 School of Software, Kunsan National University, Gunsan 54150, Republic of Korea

**Keywords:** 5G network, network slicing, machine learning, virtual network embedding, virtual network function

## Abstract

Network slicing shows promise as a means to endow 5G networks with flexible and dynamic features. Network function virtualization (NFV) and software-defined networking (SDN) are the key methods for deploying network slicing, which will enable end-to-end (E2E) isolation services permitting each slice to be customized depending on service requirements. The goal of this investigation is to construct network slices through a machine learning algorithm and allocate resources for the newly created slices using dynamic programming in an efficient manner. A substrate network is constructed with a list of key performance indicators (KPIs) like CPU capacity, bandwidth, delay, link capacity, and security level. After that, network slices are produced by employing multi-layer perceptron (MLP) using the adaptive moment estimation (ADAM) optimization algorithm. For each requested service, the network slices are categorized as massive machine-type communications (mMTC), enhanced mobile broadband (eMBB), and ultra-reliable low-latency communications (uRLLC). After network slicing, resources are provided to the services that have been requested. In order to maximize the total user access rate and resource efficiency, Dijkstra’s algorithm is adopted for resource allocation that determines the shortest path between nodes in the substrate network. The simulation output shows that the present model allocates optimum slices to the requested services with high resource efficiency and reduced total bandwidth utilization.

## 1. Introduction

The 5G network intends to enable a wide range of applications in industry, including smart security, telemedicine, home automation, augmented and virtual reality, etc. [1]. In 5G, the International Telecommunication Union (ITU) has recognized three major network services: massive machine-type communications (mMTC), enhanced mobile broadband (eMBB), and ultra-reliable low-latency communications (uRLLC). The eMBB requires a high bandwidth, as well as dependable broadband communication and low latency. For the mMTC, however, continuous wireless coverage across numerous devices is an essential requirement. The URLLC services provide very reliable, minimal latency data transfers of relatively small loads from a limited set of ports.

**Figure 1:**
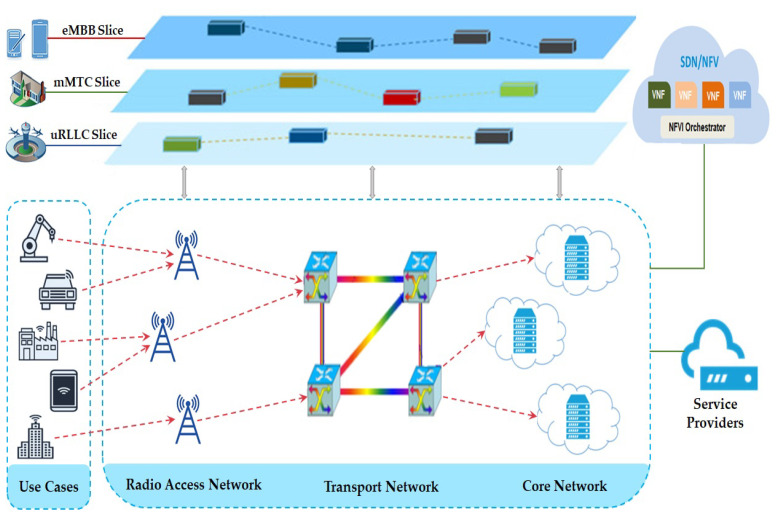
An overview of the E2E network slicing architecture.

For business applications with stringent latency specifications that need to be built on a common infrastructure, network slicing is an essential aid since it provides an efficient means of meeting the varying needs of every kind of application. The network slices could be autonomously managed, completely separate from one another, and instantiated on demand [2]. Network slicing is envisioned as a virtual network that may support numerous autonomous commercial operations in the Next Generation Mobile Networks (NGMN) [3]. Sliced networks are logically separated E2E virtual networks that can be independently managed and controlled to allow for more flexible and efficient service provision on a common physical infrastructure. Combining two emerging technologies, software-defined networking (SDN) and network function virtualization (NFV), allows for the decomposition of traditional monolithic and exclusive network applications into a large number of small, software-based, flexible networks known as virtual network functions (VNFs), which run on regular servers, thereby increasing both flexibility and efficiency [4]. NFV is well-suited for overseeing the life cycle of network slices and orchestrating VNFs, whereas SDN is best suited for configuring and regulating the forwarding planes’ initial resources [5]. Network slicing is the procedure of pooling together several VNFs. In network slicing, distributing the virtual network’s resources across the substrate network is an issue. Virtual network embedding (VNE) implies a process whereby a virtual network request (VNR) node is linked to underlying networks [6]. Figure 1 depicts the network slices as E2E logical connections that are autonomous from one another, have their own management, and can be constructed based on the requirements of the users.

To make a smart decision on the slicing, operation, and management of slicing, a massive amount of composite data must be examined so that it can reliably satisfy a service’s QoS requirement [7]. An adaptive fog configuration approach was developed by [8] for use in industrial IoT systems. This work mostly ignored the importance of allocating resources for wireless communications while focusing solely on resource management. Recent studies on combined methods such as resource allocation and management have been undertaken to further enhance the quality of service [9,10]. The act of creating network slices while concurrently analyzing massive volumes of data presents a difficult task for humans. Therefore, there is a requirement for the automation of these processes. One of the best ways to automate network slicing functions is via machine learning (ML) approaches [11]. Machine learning has been found to be effective at classifying services. ML has been found to be effective at performing service classification [12], preservation [13], security [14], prediction [15], authentication [16], and anomaly detection [17]. In particular, it can examine massive data in a short length of time, which improves the system’s ability to adapt to dynamic surroundings and make accurate predictions [14]. Because of their efficiency, robustness, self-organization, and high level of security, ML algorithms are utilized in this scenario for the purpose of resolving the classification and allocation problem [18].

In this present study, a novel E2E network slicing strategy has been developed in order to provide efficient resource allocation with minimal total bandwidth utilization. Our proposed model’s primary contributions are as follows:

- To develop an effective method for slicing networks that makes use of the ML technique for the generation of slices and dynamic programming for the distribution of resources among the produced slices.
- Multi-layer perceptron (MLP) is adopted as the method to perform network slice classification. Also, MLP is investigated using a variety of weight optimization methods, and the most accurate classifier is that which has been selected.
- For slice creation, we conduct an exhaustive comparison of various ML algorithms including K-nearest neighbor (KNN), naive Bayes, support vector machine (SVM), random forest (RF), and MLP.
- We show that the present model is superior to the existing VNE approaches by providing empirical simulation results that are run under a variety of substrate network operating conditions.

This article is structured as follows: Section 2 contains the related works, Section 3 displays the model of the network, Section 4 shows the design of the E2E network slicing using ML, Section 5 presents the simulation outputs, and, finally, Section 6 concludes this work.

## 2. Related Works

Network slicing is a technique for managing the increasing complexity of manufacturing networks in the field of industrial communication. The process of creating and deploying network slices has been the topic of a significant amount of research. In this section, we summarize recent works related to slice creation through ML techniques and research on slice deployment in 5G.

### 2.1. Slice Creation Using Machine Learning

In [19], a Markov decision process (MDP) model has been proposed to describe dynamic network state transitions. Also presented is NFVdeep, an adaptive, online, deep reinforcement learning approach to automatically deploy SFCs for requests with varying QoS needs, in order to concurrently optimize NFV provider operation costs and maximize overall request throughput. To cope with huge discrete action spaces effectively, the authors have adopted a serialization-and-backtracking technique. The use of Artificial Intelligence (AI) and ML in 5G network slicing has made way for a solution of decision-making issues and the accurate forecasting of resource and slice grouping. ML has demonstrated its utility in a variety of applications. With the intention of attaining efficient and autonomous E2E service dependability and resource tuning, an ML-based network slicing technique was suggested to execute dynamic resource scheduling [20]. In a recent study, slice allocation using different ML algorithms in a 5G network was performed, and a better quality of service was achieved [21]. Intent-based network slicing using deep learning (DL) was designed to slice and manage a core (CN) and radio access network (RAN) with minimal latency and bandwidth requirements [22]. By employing deep neural networks, 5G network slicing was able to regulate traffic on the network and route it to the most suitable slice. The DL model method was also utilized to categorize the precise network slices for each device based on their topological properties. The glowworm swarm optimization algorithm is used to adjust the weight function of the networks [18]. In a recent study, network availability and load efficiency were the focus of the deepSlice method, which made use of neural networks. Researchers have trained a system to analyze inbound connections and forecast the slice for an unknown device type using a set of key performance indicators (KPIs) [23]. However, the literature shows that ML techniques are expensive to train due to the complexity of datasets and topological features. Furthermore, some ML-based network slicing approaches continue to struggle with accurate classification. As a result, it is required to prepare the appropriate dataset with relevant KPIs and to locate the suitable ML algorithm to make network slices.

### 2.2. Slice Deployment Using Virtual Network Embedding

The initial 5G network slice architecture has been provided in [24], which proposed a time-slot base and combined edge and core cloud servers for network slicing. It utilized a demand–supply model to measure the VNF interference since VNF consolidation could result in a significant decline in performance. The process of deploying a number of slices is one of the most challenging tasks associated with network slicing [25]. Most network slicing deployments have occurred in the fixed core, while E2E network slicing in 5G is still evolving. The PERMIT slice orchestration system is presented in [26], and it is the very first system to take E2E slicing into consideration. Embedding the virtual network node slices into the actual physical networks is the primary obstacle to deploying E2E network slicing. This virtual network is made up of VNFs, which together form the vertical’s serving system. Embedding comprises two procedures, node and link mapping, which are concerned with the assignment of virtual nodes and links to substrate nodes and links [27]. The VIKOR algorithm, a multi-criterion decision-making method, was used to create a provisioning procedure for 5G network core slices. The VIKOR method is used to categorize node significance in the node slice provisioning step by taking topological attributes and network resources into account. In another study, to increase the slice acceptance ratio, the K-shortest path algorithm was utilized to find the shortest routes between candidates in the link [28]. The breadth-first search technique was also used to identify the order of node and link embedding at the stages of the node embedding procedure [29]. Complex network theory was employed to accomplish E2E slicing, with the resulting model running on topological knowledge acquired beforehand. During link mapping, the Floyd method was utilized to determine the shortest path between the nodes [30]. According to the studies cited in Table 1, a significant amount of investigation has been conducted into the processes that are used for the creation and deployment of slices. The majority of the works in the table provide methods for slice creation [21,22,23], whereas others are exclusively concerned with slice isolation [27,28,29,30]. However, integrating the slice creation and deployment processes is essential for measuring the efficacy of E2E network slicing. In our proposed work, ML is used to create slices and dynamic programming is used in their deployment. As an added measure, we employed scenario-specific metrics to assess how well the suggested method performs.

## 3. Network Model

The substrate network (SN), virtual network request (VNR), and slice allocation models make up the proposed network model, which is described in detail below.

### 3.1. Substrate Network

The topological properties of the SN, representing the structural characteristics of actual nodes (including base stations (BS), optical switches (OS), and CN), are included in the E2E network-slicing framework. The SN is depicted by an undirected weighted scale-free network graph, denoted as $G^{S}=\left(N^{S},E^{S}\right)$. The collection of SN nodes, denoted by $N^{S}$, can be further split into $N_{BS}^{S}$, $N_{OS}^{S}$, and $N_{CN}^{S}$, $N^{S}\in N_{BS}^{S}\cup N_{OS}^{S}\cup N_{CN}^{S}$. Each substrate node supplies the following resources: $N_{Res}^{S}\left(C^{S},LC^{S},D^{S},SL^{S}\right)$. $C^{S}$ denotes the CPU capacity, $LC^{S}$ represents link capacity, $D^{S}$ denotes delay, and $SL^{S}$ represents the security level. $E^{S}$ denotes a set of links in the SN. The available bandwidth for the link $E^{S}$ is denoted by $B^{S}$.

### 3.2. Virtual Network Request

Each VNR includes eMBB, mMTC, and uRLLC requests that can be denoted as $VNR\in VNR_{e}\cup VNR_{m}\cup VNR_{u}$. Each VNR is denoted as an undirected graph $G^{V}=\left(N^{V},E^{V}\right)$; here, $N^{V}$ represents the VNR node. Each VNR node has a set of requirements of $N_{Req}^{V}\left(C^{V},LC^{V},D^{V},UT^{V},SR^{V}\right)$. $C^{V}$ denotes CPU capacity, $B^{V}$ denotes bandwidth, $LC^{V}$ denotes link capacity, $D^{V}$ denotes delay, $UT^{V}$ denotes user type, and $SR^{V}$ represents the security level. $E^{V}$ denotes a set of links in the VNR. The bandwidth for the link $E^{V}$ is denoted by $B^{V}$.

### 3.3. Slice Allocation

Slice allocation is the method of mapping VNR nodes and links onto SN nodes and links to meet slice service requests. The node allocation process is defined as the function $f_{NM}$ : $N^{V}\to N^{S}$, which maps a request node to an SN node. The link allocation function is represented as $f_{LM}$ : $E^{V}\to E^{S}$.

We created the resource allocation method for user equipment (UE), with the aim of minimizing the total bandwidth utilization while guaranteeing a higher user access rate and efficiency. So, it can be formulated as the resource allocation problem as follows:

(1) $$min\sum_{i\in N^{S}}\sum_{m\in N^{V}}x_{i}^{m}C_{i}^{V}+\sum_{ij\in E^{S}}\sum_{mn\in E^{V}}w_{ij}^{mn}B_{mn}^{V}$$

(2) $$subject\;to,\sum_{i\in N^{S}}\sum_{m\in N^{V}}x_{i}^{m}C_{m}^{V}\leq C_{i}^{S}$$

(3) $$\sum_{ij\in E^{S}}\sum_{mn\in E^{V}}w_{ij}^{mn}B_{mn}^{V}\leq B_{ij}^{S}$$

(4) $$\sum_{i\in N^{S}}\sum_{m\in N^{V}}x_{i}^{m}\left(d_{m}^{V}+D^{i}\right)\leq d_{i}^{S}$$

(5) $$\sum_{i\in N^{S}}\sum_{m\in N^{V}}x_{i}^{m}=1$$

(6) $$x_{i}^{m}\in 0,1,\forall \left(i,m\right)\in \left(S,V\right),$$

where $x_{i}^{m}$ and $w_{ij}^{mn}$ are the binary variables. $x_{i}^{m}$ indicates that the $m^{th}$ request node is served onto the $i^{th}$ substrate node, and $w_{ij}^{mn}$ indicates that the substrate link ij hosts the virtual link mn. If the $i^{th}$ node of $N^{V}$ is provisioned onto the $m^{th}$ node of $N^{S}$, then $x_{i}^{m}$ is one; otherwise, it is zero. If the substrate link ij hosts the virtual link mn, then $w_{ij}^{mn}$ is one; otherwise, it is zero. Constraint (2) ensures that the CPU capacity of a request never exceeds the CPU capacity of its SN node. Constraint (3) ensures the bandwidth allocated for a request does not exceed the maximum value $B_{ij}^{S}$. Constraint (4) guarantees the delay of the VNR, which can be satisfied by the delay of the SN. Constraints (5–6) ensure that each node can access only one VNR via one SN at a time. According to this constraint, resources are allocated for the received virtual request with the minimum total bandwidth consumption.

## 4. Design of E2E Network Slicing Using Machine Learning

**Figure 2:**
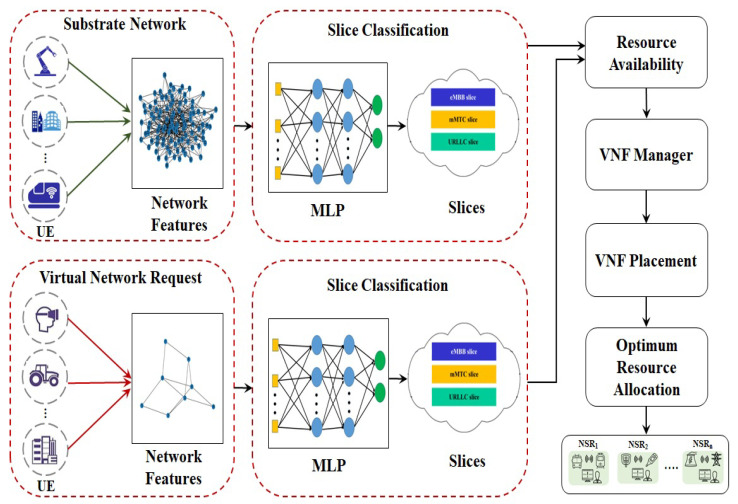
Proposed model for slice creation and deployment.

Network slicing divides an actual network into numerous E2E logical connections that are self-managed and produced on demand. Massive data analysis in a short amount of time is required in 5G. The analysis of massive volumes of information and accurate slice prediction could be made possible by the implementation of a suitable ML algorithm. Our work primarily contributes to providing optimal 5G network slicing by using MLP to accomplish high accuracy in classification. Also, we extended our work to provide optimum resource allocation through dynamic programming. Figure 2 shows the design of the E2E network slicing strategy through MLP. At first, it is necessary to notify a slew of different UE devices operating in the 5G network. In order to perform classification analysis, the UEs with various KPIs, such as CPU capacity, bandwidth, delay, link capacity, security level, and user type, are extracted. Slice creation using MLP with the ADAM function has been attempted in order to maximize classification accuracy. The nodes are categorized into eMBB, mMTC, and uRLLC based on the information they receive from MLP. After slice prediction, the VNF manager allocates resources based on the available resources. The VNF placement involves node mapping and link mapping to ensure the most efficient use of the available resources. Node mapping uses a sequential algorithm to allocate nodes to each request. Using a dynamic programming method, link mapping produces an optimal allocation with the smallest possible link utilization. The shortest path between the nodes is selected using Dijkstra’s algorithm, which reduces the overall bandwidth cost.

### 4.1. Machine Learning-Based Slice Classification Model

We utilize supervised learning methods to handle efficient slice prediction, network slice failure, and optimal resource usage. Various supervised learning algorithms, such as KNN, naive Bayes, SVM, RF, and MLP are investigated for our network features. Based on performance metrics, the most suitable learning scheme is identified to perform classification. Algorithm 1 shows the activity for node slice classification using MLP.
**Algorithm 1** Slice classification using MLP**Input:** Substrate network $G^{s}\left(N^{s},E^{s}\right)$**Output:** Node slice classification1:Set up *eMBB*, *mMTC*, *uRLLC* to be a blank *k*-length vector.2:Set node i=03:Get KPI for $N^{s}$4:Perform classification: **MLP Classifier()**5:**while** i<=n **do**6:   $X_{Pred}$ of node *i*7:   Y${}_{Pred}$ = Predict the class of X${}_{Pred}$8:   **if** Y${}_{Pred}$ = "eMBB Class" **then**9:     *eMBB* = *i*
10:  **else if** $Y_{Pred}$ = "mMTC Class" **then**11:    *mMTC* = *i*12:  **else**13:    *uRLLC* = *i*14:  **end if**15:  *i = i + 1*16:**end while**17:*eMBB, mMTC, uRLLC* nodes

The efficacy of the supervised categorization models is measured in four different ways. The cases that are successfully labeled as positive by the model are called true positives (TP), whereas those that are wrongly labeled as negative are called false negatives (FN). Similarly, genuine negative cases that the model correctly marks as negative cases are referred to as true negative (TN) cases, while actual negative cases that the model incorrectly marks as positive cases are referred to as false positive (FP) cases. The procedures for calculating performance measures [31], including accuracy (AUC), precision, recall, and F1 Score, are

(7) $$Accuracy=\frac{TP+TN}{TP+FP+FN+TN}$$

(8) $$Precision=\frac{TP}{TP+FP}$$

(9) $$Recall=\frac{TP}{TP+FN}$$

(10) $$F1\;Score=2*\frac{Precision*Recall}{Precision+Recall}.$$

A labeled data collection is employed to train the model in the supervised learning approach. The system is aware of both the input data and the supervised output data that must be created. The following section will go through the various supervised learning algorithms [32,33] considered in this proposed model to perform classification.

#### 4.1.1. K-Nearest Neighbor

The KNN algorithm is a classic instance of a non-parametric classification approach because it does not presuppose anything about the training data. Using a labeled training dataset, where points have been assigned to several classes, it is possible to predict the class of unlabeled data. The unknown tuple can be classified using *K*’s fixed value. When KNN discovers a novel unlabeled tuple in the dataset, it performs two actions. First, it finds its *K*-nearest neighbors or points that are immediately adjacent to the new data point. The second part of KNN is that it uses neighboring data to figure out what category the new information belongs in. The Euclidean distance should be used to calculate the distance between the test sample and the specified training samples. The Euclidean distance function is as follows:

(11) $$\sqrt{\sum_{i=1}^{m}{\left(x_{i}-y_{i}\right)}^{2}}.\;$$

#### 4.1.2. Naive Bayes

The naive Bayes method is an approach to data classification that makes use of the probabilistic values provided by the Bayes theorem, as well as the assumption of naive independence. It calculates the probability of each class in a dataset, returns the value P(Y|X) , and uses discriminative learning to make predictions about the property of the new class. The naive Bayes classifier is formally defined as follows. Let *N* stand for the training set, which comprises the class labels, and each set element *X* is a vector value of $X=x_{1},\;x_{2},\;x_{3},\ldots ,\;x_{n}$. Let us say there are *S* classes and the class labels are $Y=y_{1},\;y_{2},\;y_{3},\ldots ,\;y_{s}$. The classifying amount optimizes the probability P(Y|X) to identify the most evidence of X in any of the Y classes if an attribute vector *X* occurs. The Bayes theorem yields the (P(Y|X) as

(12) $$P\left(Y|X\right)=\frac{P(X|Y)\times P(Y)}{P(X)},$$

where *P(Y)* is the a priori probability. The naive Bayes assumption is independent for the attributes $x_{1},\;x_{2},\;x_{3}\ldots ,\;x_{n}$. So,

(13) $$P\left(Y|X\right)=P\left(x_{1}|Y\right)\times P\left(x_{2}|Y\right)\times P\left(x_{3}|Y\right)\ldots \times P\left(x_{n}|Y\right).$$

#### 4.1.3. Support Vector Machine

With proper training, support vector machines (SVMs) can detect the hyperplane that effectively divides a dataset into two classes, allowing for the classification of data points into one of two groups [34]. The SVM creates a model from the data in the training set. The model is then utilized to classify specific instances within the test data after it has been constructed. Consequently, the SVM locates the hyperplane that divides the data into its two classes in the best possible way. The SVM hyperplane is denoted as

(14) $$h_{w,b}\left(x\right)=g\left(w^{2}x+b\right),$$

where *w*, *x*, *b* refer to weights, input, and bias, respectively.

#### 4.1.4. Random Forest

The RF classifier is a collective learning algorithm that uses a fixed number of trees to classify and predict outputs. We need to process *n* texts, where n is the total number of trees in the dataset. An optimal split requires classifying each record in the dataset at random among all the predictor classes. When *m* (tries) = *P*, a specific state named bagging can be learned. Unlabeled classes should be predicted using a collected number of aggregate prediction trees. RF is made up of a set of tree-structured classifiers $h(x,\theta_{k}),\;k=1,\;2,\ldots$, where *k* is a set of identically distributed random vectors. The prediction error (PE) of RF is given as

(15) $$PE=P_{x,y}\left(mg\left(X,Y\right)\right).$$

Here, mg(X,Y) is the margin function. The margin function calculates by how many votes the correct class at *(X, Y)* is preferred over the other classes. In this case, *X* is the predictor vector and *Y* is the classification. The following equation represents the margin function:

(16) $$\begin{array}{c} mg\left(X,Y\right)=av_{k}I(h_{k}\left(X\right)=Y)-max_{j\neq Y}\;\\ av_{k}I\left(h_{k}\left(X\right)=j\right). \end{array}$$

#### 4.1.5. Multi-Layer Perceptron

**Figure 3:**
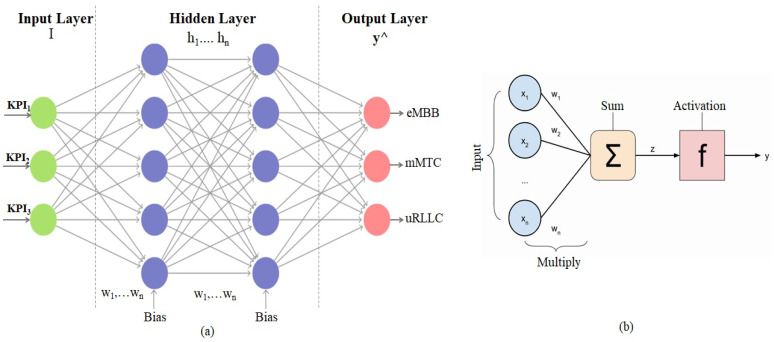
(**a**) Structure of MLP. (**b**) Neurons in MLP.

The design of an artificial neural network (ANN) is based on layer connectivity through nodes referred to as neurons, which are analogous to biological neurons in the human brain. Each path transfers a signal between layers in the same way as terminals. The multi-layer perceptron (MLP) is a feedforward neural network that has three layers: an input layer, one or more hidden layers, and an output layer. Figure 3a depicts the structure of the proposed MLP. The KPI of user devices is given as an input, two hidden layers are taken into consideration, and 20 neurons are taken for each hidden layer. The neuron cell output values can be adjusted using weighted factors.

Stochastic gradient descent (SGD), limited-memory broyden fletcher goldfarb shanno (LBFGS), and ADAM are only a few of the techniques used to optimize weights [35]. Multiplying the link weights by the neuron output values via the additive operator yields the cumulative input. The cumulative input also includes the bias value, which is applied to it. The activation function processes the cumulative input value obtained from the additive function to produce the desired result for the neuron. The sigmoid activation function is used, which is a non-linear, elementary-derivative-based continuous function. The complete process of a neuron in the MLP is shown in Figure 3b. *N* neurons make up the input layer and *M* neurons make up the hidden layers of an MLP. The number of KPIs $x_{i}$ and a set of equivalent weights $\left(w_{ij}\right)$ are used to compute the outputs of all neurons in the hidden layer. The output neurons ($O_{i}$) of the hidden layers are calculated using

(17) $$O_{i}=\sum_{i=0}^{N}w_{ij}x_{i}$$

(18) $$y_{j}=z\left(O_{j}\right),$$

where i=1,2,…,N and j=1,2,…,M. The sigmoid activation function is represented by *z*, and the hidden layer’s $j^{th}$ node’s output is denoted by $y_{j}$. *z* is represented as

(19) $$z\left(x\right)=\frac{1}{1+e^{-x}}.$$

Each neuron in the output layer sends its final signal as

(20) $$y^{\wedge }=f\left(\sum_{j=0}^{m}w_{j}y_{j}^{H}\right),$$

where the activation function is *f* and $y^{\wedge }$ is the output of the MLP. The MLP always tries to keep the error as small as possible through the Back Propagation (BP) training algorithm. Different weight optimization functions, including ADAM, SGD, and LBFGS, have been tested and report the best weight optimization function for slice classification. In SGD, instead of using the complete dataset for each iteration, it chooses small batches of data at random in each iteration step. That means it only takes a small sample of the dataset. As a result, SGD requires more iterations to reach the local minima. LBFGS can converge faster and perform better on small datasets. However, it does not produce better results for larger datasets. The ADAM optimizer outperforms all other optimization algorithms in terms of overall performance, computational cost, and tuning parameters. Based on performance outcomes, the ADAM optimizer is selected in MLP to classify our network slices.

### 4.2. Resource Allocation for E2E Network Slicing Using Dynamic Programming

**Figure 4:**
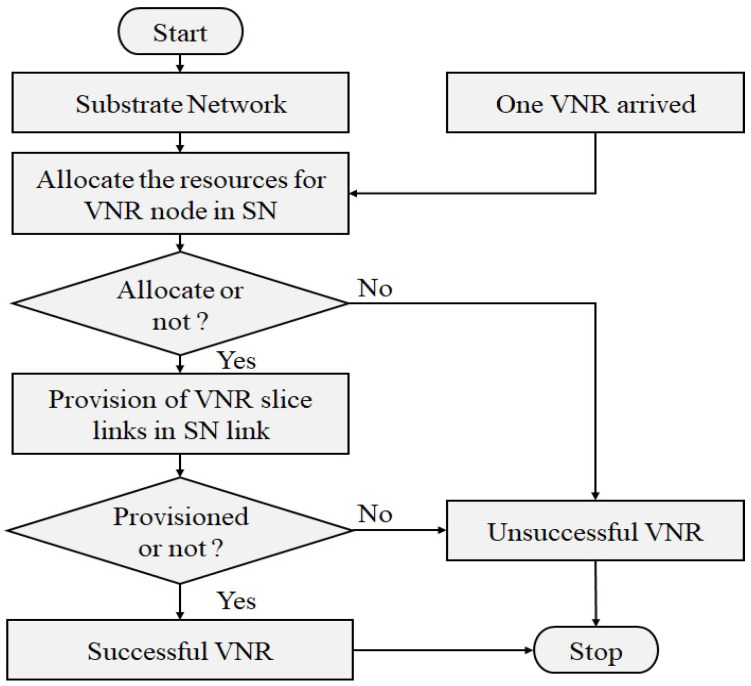
Flowchart of resource allocation for one VNR.

This section presents a dynamic programming method that is used to solve the resource provisioning problem in 5G, which includes the node provisioning and link provisioning stages. In the process of node provisioning, the resources that are required by the request nodes are supplied by the SN nodes. If the process is completed, then the request will be transferred to the process of link provisioning. Otherwise, the VNR will fail. The request links are served by the SN links during the link provisioning process. If it is successful, the VNR will also be successful (sVNR). If not, the VNR will fail. The steps that go into allocating resources for the VNRs are outlined in Figure 4, which offers a graphical representation of those steps. The main aim of the service provisioning process is to minimize the total bandwidth utilization and improve the user access rate with high resource efficiency.

#### 4.2.1. Resource Provisioning Framework

When a virtual request is received by the service provisioning system, the mechanisms of node and link mapping are used to offer the required resources. A sequential array-based technique is utilized in the process of node mapping to assign the virtual request nodes to the SN. During the process of node allocation, it is important to check that the substrate node satisfies the resource limitations and security constraints imposed by the virtual nodes. If it satisfies the constraints, then the virtual request nodes are successively hosted on the substrate nodes; otherwise, the service is not provided to the virtual request. In link mapping, the dynamic programming based on Dijkstra’s shortest path algorithm is implemented in the substrate connections in order to facilitate the provisioning of virtual request links. The Dijkstra algorithm is used in the process of locating the most efficient route in the SN for virtual request links. Then, we check whether the substrate links satisfy the constraints of the virtual request links or not. If it satisfies the constraints of the virtual links, then the substrate network provides the service to the virtual request links; otherwise, VNR fails. The two stages of the resource allocation procedures are described in Algorithm 2.
**Algorithm 2** Resource allocation through dynamic programming**Input:** SN: $G^{s}$, VNR: $G^{V}$**Output:** $G^{s},sVNR$1:Receive the *VNR* $G_{k}^{V}$ nodes with *KPI*2:Arrange the $G_{n}^{S}$ and $G_{k}^{V}$ nodes in sequential order3:**for** each VNR node **do**4:   Nodes in $G_{k}^{V}$ and $G_{n}^{S}$ are arranged in descending order based on value5:   Slice nodes of $G_{k}^{V}$ are allocated into SN nodes $G_{n}^{S}$6:   **if** slice nodes allocation failed **then**
7:     **return** *usVNR*8:   **else**9:     Check constraints for link provisioning10:    **for** each VNR link ($L^{V})$ **do**11:       Determine the required bandwidth B(L)12:       Remove the substrate links that fall short of the required bandwidth13:       Using the mapping node, identify the mapped SN nodes of *L*.14:       Apply Dijkstra’s algorithm to find the shortest path between these two nodes15:       **if** link (*L*) is present **then**16:         Slice links $L^{V}$ are provisioned into SN link $L^{S}$17:         Update $G^{S}$18:         **return** *sVNR*, $G^{S}$19:       **else**20:         Slice links $L^{V}$ are not provisioned into SN link $L^{S}$21:       **end if**22:    **end for**23:  **end if**24:**end for**

#### 4.2.2. Performance Metrics for Optimal Resource Allocation

The effectiveness of the slice allocation method is measured here in terms of user access rate and resource efficiency in terms of minimum total bandwidth consumption. The metrics are defined as follows:

*(i)*  *User access rate (UAR):*
  (21) $$UAR=\lim_{T_{max}\to \infty }\frac{\sum_{t=0}^{T_{max}}sVNR\left(t\right)}{\sum_{t=0}^{T_{max}}TVNR\left(t\right)},$$
  where sVNR is the number of VNRs that are allocated into SN successfully and TVNR is the total number of requests received from time 0 to $T_{max}$.
*(ii)*  *Resource efficiency (RE):*
  (22) $$RE=\frac{\sum_{n\epsilon N^{V}}C^{V}\left(n\right)+\sum_{L\epsilon E^{V}}B^{V}\left(L\right)}{\sum_{n\epsilon N^{V}}C^{V}\left(n\right)+\sum_{L\epsilon E^{V}}B^{V}\left(L\right)\times hop\left(L\right)},$$
  where $C^{V}\left(n\right)$ shows the CPU load of the virtual node *n*, $B^{V}\left(L\right)$ denotes the bandwidth of the virtual link *L*, and hop(L) is the mapping length of the virtual link *L*. The revenue–cost ratio is used to calculate the efficiency of resources. A substrate network’s revenue from adopting a VNR is defined as the sum of the VNR’s capacity and link bandwidth requirements. The cost of a resource is determined by adding up the capacity of the nodes, the bandwidth resources of the link, and the path length of the link provided by the SN for the corresponding VNR nodes.
*(iii)*  *Node utilization (NU) and link utilization (LU):*
  (23) $$NU=\sum_{n=1}^{TVNR}TC^{V}\left(n\right)$$
  (24) $$LU=\sum_{n=1}^{TVNR}TB^{V}\left(n\right),$$
  where TVNR denotes total VNR, $TC^{V}\left(n\right)$ represents the total CPU capacity utilized for a VNR, and $TB^{V}\left(L\right)$ denotes the total bandwidth utilized for a VNR.

## 5. Experimental Results and Analysis

In this work, the developed model’s classification accuracy, resource efficiency, and access rate are validated. The proposed model is implemented in Python and its performance analysis has been conducted. First, we describe the setting of our experiments under various testbed parameters and then show the simulation results to demonstrate the accuracy of the slice classification. Finally, we analyze the generated simulation results and show the outcome of resource efficiency, access rate of users, and resource utilization.

### 5.1. Simulation Testbed Settings

In the beginning, an undirected graph with a varying number of nodes is constructed for both the SN and the VNR. The Barabasi–Albert scale-free network algorithm [36] is applied to create an undirected graph. The total number of nodes considered for evaluation in the SN was $N^{S}$ = 100, 200, 300. Every VNR consists of 20 nodes. Both the number of requests and the LT of requests can vary ranging between 5 and 40 units. For evaluation, 5 to 40 VNR are taken into account, and each VNR has 20 nodes, so a total of 100 to 800 nodes are taken into consideration. Every node in the network has its own unique set of topological properties [30,37], which may include CPU power, bandwidth, link capacity, delay, and level of security. The KPIs of the SN and VNR are represented in Table 2.

### 5.2. Performance Evaluation

To assess the effectiveness of the developed model, we employ three different measures: classification accuracy, resource efficiency, and user access rate. A dataset with 1000 samples has been prepared for evaluation. The dataset is divided into three categories, including eMBB, mMTC, and uRLLC. In order to perform analysis, separate datasets have been constructed for both the SN and VNR.

#### 5.2.1. Classification Reports

Table 3 shows the slice classification and accuracy reports for MLP with various optimizers. The table shows that the ADAM optimizer performs the most accurate classification among all the optimizers. As a result of this, in order to facilitate additional research, ADAM-based MLP classification has been taken into consideration for the purpose of comparing its performance with that of other machine learning algorithms. When comparing the effectiveness of various training techniques, precision, recall, and F1 Score measures for the eMBB, mMTC, and uRLLC slices are considered.

**Figure 5:**
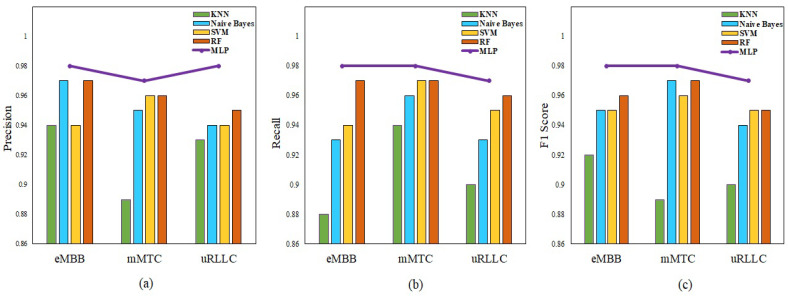
Performance measurements: (**a**) precision, (**b**) recall, and (**c**) F1 Score.

Figure 5 illustrates the performance of different machine learning methods. All of these methods are capable of achieving a result that is greater than 0.88. For all slices, the MLP using the ADAM approach obtains a score of above 0.97 for precision, recall, and F1 Score.

**Figure 6:**
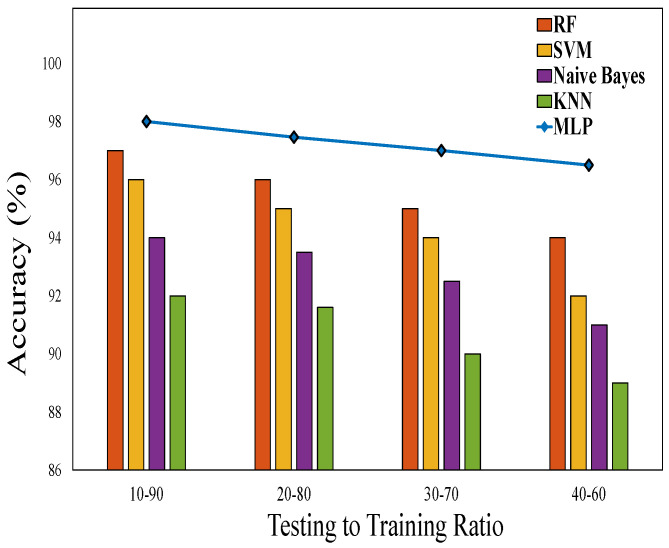
Classification accuracy.

Slice classification with various training–testing ratios of 9:1, 8:2, 7:3, and 6:4 is shown in Figure 6. The proposed MLP provides better accuracy for the 5G network slicing process compared to other machine learning approaches. In the case of dynamic deployment, each set of VNRs has 20 nodes equipped with eMBB, mMTC, and uRLLC slices.

#### 5.2.2. Performance of User Access Rate and Resource Efficiency

**Figure 7:**
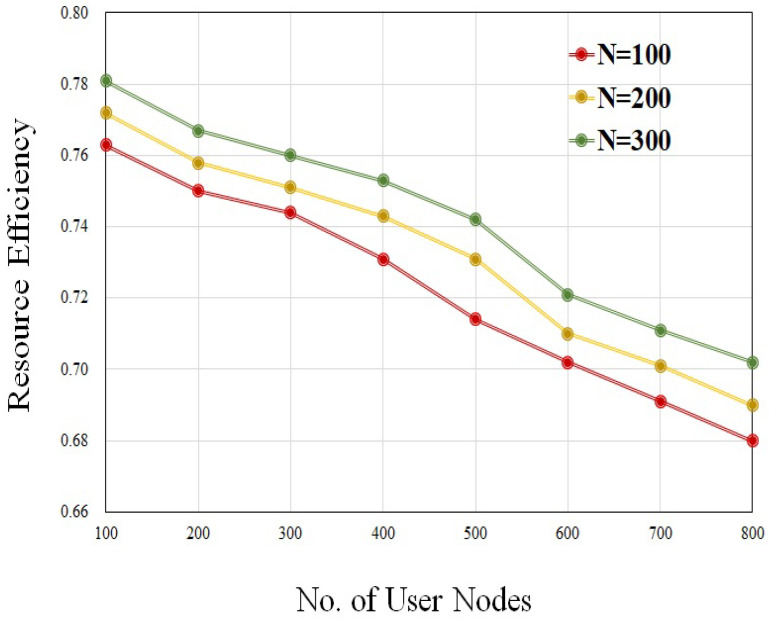
Resource efficiency with different SN nodes.

**Figure 8:**
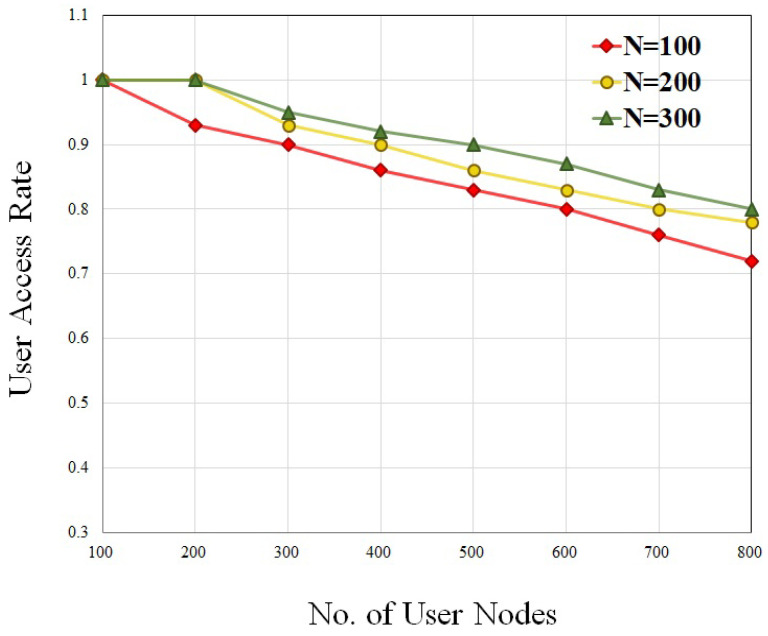
User access rate with different SN nodes.

The effectiveness of slice deployment is measured by utilizing metrics such as resource efficiency and access rate. In order to ensure that there is an adequate supply of substrate resources, we calculate the resource efficiency with the SN nodes, N = 100, 200, 300, and user request nodes ranging from 100 to 800. Figure 7 shows the result of resource efficiency under different system operating conditions. According to this figure, the efficiency of the resources drops as the number of user request nodes increases. And, it is shown that the efficiency of the resources is increased when the number of nodes in the SN grows. Intuitively, the user access rate is directly proportional to the number of slices that have been successfully deployed. In the condition of dynamic deployment, we conduct an analysis of the relationship that exists between the total number of user request nodes and the user access rate. Figure 8 shows the result of the user access rate at different request user nodes. When the time duration is prolonged, the resources are not frequently updated, which leads to a reduction in AR.

**Figure 9:**
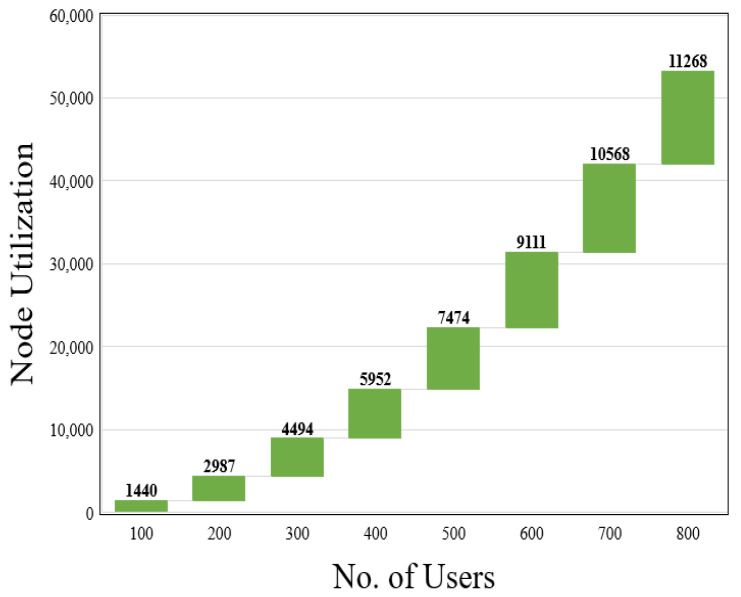
Node utilization of proposed system.

**Figure 10:**
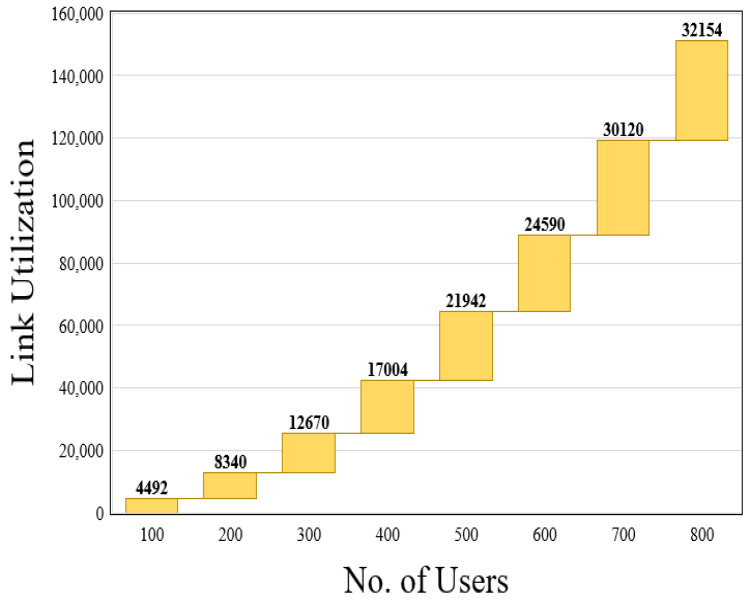
Link utilization of proposed system.

The results of the resource utilization are shown in Figure 9 and Figure 10. Our primary goal is to reduce total bandwidth utilization by employing Dijkstra’s shortest path algorithm and achieve a higher user access rate while conserving resources. When the number of users grows, our scheme significantly improves the resource utilization. Figure 9 depicts the node utilization of our scheme with varying user counts. Figure 10 represents the outcome of our method’s link utilization with a diverse set of users. When comparing the results of resource efficiency for different VNRs, the proposed procedure achieves a resource efficiency of 0.79 for a given number of substrate nodes.

**Figure 11:**
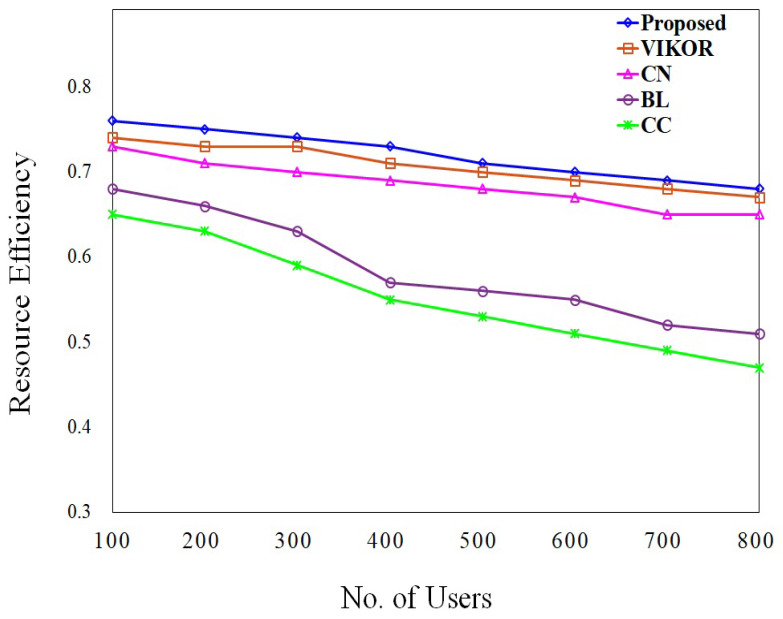
Resource efficiency comparison with existing algorithms.

Compared to the existing algorithms such as the VIKOR [28], CN [30], BaseLine (BL) [38], and Closeness Centrality (CC) [39] methods, the efficiency results achieved by the proposed algorithm are marginally superior. Due to the incorporation of ML into our classification method, the classification accuracy of the nodes is automatically improved. Along with it, we implemented VNE with a dynamic programming algorithm, which subsequently led to a reduction in the overall utilization of the network bandwidth. Figure 11 presents the findings regarding the efficiency of the used resources.

**Figure 12:**
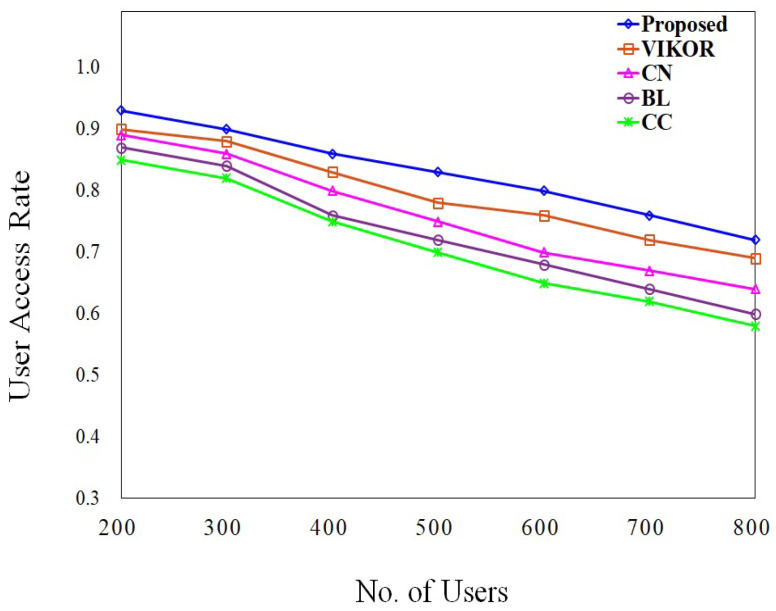
User access rate comparison with existing algorithms.

The number of successful requests is applied to compute the UAR. The comparison of the proposed UAR with other algorithms is shown in Figure 12. This graph shows that the access rate of the existing algorithms is decreased when the number of VNRs and the LT of each VNR is increased. On the other hand, when the number of VNRs is modest, the proposed algorithm yields similar results to the VIKOR method.

However, when the number of VNRs rises, the proposed technique performs better than the other strategies. The UAR of CN is higher than the BL and CC methods. As a result, as the number of VNRs grows, the proposed method produces better outcomes than all other algorithms. When the number of users grows, more resources must be allocated to the service, so the UAR will gradually decrease.

## 6. Conclusions

In this work, we developed an efficient technique for E2E network slice creation and service provisioning using an ML algorithm, which includes two main phases: (a) slice classification using the supervised learning method and (b) optimum resource allocation using Dijkstra’s algorithm. First, we constructed an undirected graph for both SN and VNR that includes various network features, such as CPU capacity, bandwidth, user device type, link capacity, delay rate, and security level. After that, we applied various ML techniques to perform slice classification and found the most effective method for our network features. And, we adopted MLP with the ADAM optimizer to classify each node in SN and VNR into the exact network slices, such as eMBB, mMTC, or uRLLC. The simulation model and analytical findings demonstrate that the MLP model has the potential to give accurate network slice classification in 5G. After classification, optimum resource allocation was performed by using the dynamic programming strategy. With Dijkstra’s algorithm, the shortest path between nodes is calculated, which minimizes the total bandwidth use of the network. Our suggested algorithm’s performance has been verified through extensive simulations, and the resource usage has been examined. The experimental outcomes demonstrate that the proposed algorithm achieved a higher user access rate and resource efficiency at dynamic LT. Hence, our proposed E2E network slicing scheme performs better in terms of accuracy, access rate, and resource efficiency compared to existing algorithms. Furthermore, the generated model will be evaluated on numerous other datasets. Any other methodology for map nodes will be found to improve efficiency as well. 

## Figures and Tables

**Table 1:** Summary of the literature.

Ref. No.	Methodology	Techniques	Objectives	Limitations
[21]	Supervised machine learning	K-nearest neighbors Random forest	- To meet QoS and SLA, allocates slices for services through ML	- Works better with the dataset used, but its performance measurement is not performed under high traffic
[22]	Deep learning	Generative adversarial neural network	- To automate slice creation and network settings	- Limited functionalities and need to add more features for network slicing life cycle management
[18]	Machine and deep learning	Deep neural network Glowworm swarm optimization and deer hunting optimization algorithm	- To provide accurate network slicing	- Used for small-scale problems, but has to be performed for real-time data and a huge amount of data in the cloud
[23]	Deep learning	Deep learning neural network	-To efficiently handle network load balancing and slice failures	- Due to the complexity of the dataset, training is incredibly expensive
[27]	Shareable VNF	Integer linear program Back-tracking coordinated virtual network mapping	- To minimize the resource consumption by integrating shareable VNF instances to increase acceptance rate and efficiency	- Assumed as all nodes of NSR requests for similar kinds of use cases.
[28]	VIKOR approach	Integer linear program Multi-criteria decision making	- To maximize slice acceptance and provide revenue-to-cost	- Performed better in slice traffic loads but need to improve performance in dynamic reprovisioning of slices
[30]	Complex network theory	Integer linear program Floyd algorithm	- To achieve higher resource efficiency and acceptance ratio with minimum execution time	- Assumed as all nodes of NSR requests for similar kinds of use cases

**Table 2:** Simulation parameters.

Substrate Network	
Terms	Specifications
$C^{S}$	Each node contains 20 to 50 CPU units
$LC^{S}$	Each node has a 20 to 50 unit link capacity
$SL^{S}$	The security level of a node from 0 to 1
$B^{S}$	Bandwidth of each link in the range of 20 to 50 units
$D^{S}$	Each node’s delay time varies from 0 to 1
**Virtual Network**	
Terms	Specifications
$C^{V}$	CPU capacity of each VNR node in the range of 5 to 25 units
$LC^{V}$	Link capacity of each VNR node in the range of 5 to 25 units
$SR^{V}$	Available security level of a request in the range of 0 to 0.5
$B^{V}$	Bandwidth of each VNR link in the range of 0 to 25 units
$D^{V}$	Delay time between each node is between 0 and 0.5

**Table 3:** Summary of the literature.

MLP	eMBB			mMTC			uRLLC			Accuracy
	**Precision**	**Recall**	**F1 Score**	**Precision**	**Recall**	**F1 Score**	**Precision**	**Recall**	**F1 Score**	**(%)**
LBFGS	0.89	0.92	0.89	0.88	0.85	0.89	0.88	0.91	0.88	**88**
SGD	0.90	0.95	0.91	0.98	0.97	0.98	0.92	0.83	0.87	**93**
ADAM	0.98	0.98	0.98	0.98	0.97	0.98	0.97	0.98	0.97	**98**

## Data Availability

No new data were created or analyzed in this study. Data sharing is not applicable to this article.
